# Public involvement in the Swedish health system: citizen dialogues with unclear outcomes

**DOI:** 10.1186/s12913-023-09947-x

**Published:** 2023-09-04

**Authors:** Mio Fredriksson, Anton Modigh

**Affiliations:** Department of Public Health and Caring Sciences, Health Services Research, Uppsala University, Postal address: Box 564, Uppsala, 751 22 Sweden

**Keywords:** Patient and public involvement, Citizen participation, Citizen dialogues, Swedish health system

## Abstract

**Background:**

In systems with representative democracy, there is a growing consensus that citizens should have the possibility to participate in decisions that affect them, extending beyond just voting in national or local/regional elections. However, significant uncertainty remains regarding the role of public involvement in decision-making, not least in healthcare. In this article, we focus on citizen dialogues (CDs) in a health system that is politically governed and decentralised. The aim of the study was to evaluate the functioning of citizen dialogues in the Swedish health system in terms of representation, process, content, and outcomes.

**Methods:**

This study was conducted using a qualitative case design focusing on CDs at the regional level in Sweden. The regional level is politically elected and responsible for funding and provision of healthcare. The data consist of public documents describing and evaluating the CDs and interviews, which were analysed drawing on a modified version of the Abelson et al. analytical framework for evaluating public involvement in healthcare.

**Results:**

Some CDs were an attempt to counteract political inequality by inviting groups that are less represented, while others aimed to increase legitimacy by reducing the distance between policymakers and citizens. The results from the CDs—which were often held in the beginning of a potential policy process—were often stated to be used as input in decision-making, but *how* was not made clear. Generally, the CDs formed an opportunity for members of the public to express preferences (on a broad topic) rather than developing preferences, with a risk of suggestions being too unspecific to be useful in decision-making. The more disinterested public perspective, in comparison with patients, reinforced the risk of triviality. A need for better follow-up on the impact of the CDs on actual decision-making was mentioned as a necessary step for progress.

**Conclusions:**

It is unclear how input from CDs is used in policymaking in the politically governed regions responsible for healthcare in Sweden. The analysis points to policy input from CDs being too general and a lack of documentation of how it is used. We need to know more about how much weight input from CDs carry in relation to other types of information that politicians use, and in relation to other types of patient and public involvement.

**Supplementary Information:**

The online version contains supplementary material available at 10.1186/s12913-023-09947-x.

## Background

Health systems differ in terms of funding, provision, and governance, and can be placed between free market systems and government monopoly systems [1, 2]. The health policy context in countries varies depending on a country’s social, cultural, and political fabric. A country’s political institutions and traditions are crucial, and importantly, policymaking authority in health systems can be highly centralised or dispersed at multiple levels. How formal institutions for making public policy decisions are designed also vary, as well as the distribution of power between levels of government and other actors such as unions, professional bodies, insurance providers, and the medical industry [3, 4]. In this article we focus on public involvement in a health system that is politically governed, both nationally and at the local (municipal) and regional levels. In this way, research on patient and public involvement in healthcare is interwoven with research on citizen participation in public policymaking within the framework of representative democracy. Below, an introduction to patient and public involvement and citizen participation, respectively, is provided, as well as a brief description of the empirical case—citizen dialogues in the Swedish health system.

### Patient and public involvement

Patient and public involvement (PPI) has become an important aspect of health systems during the last decades. The main arguments for PPI are, first of all, that it is morally right that the ones affected by and paying for healthcare services should have a say on implementation and delivery, and second, that it will improve the quality, efficiency, and results of healthcare [5]. PPI can be described as the “active participation of citizens, users and carers and their representatives in the development of healthcare services and as partners in their own healthcare” [6]. Hence, PPI covers both patients and the public, and according to some researchers, there are important differences between the two roles, with particular expectations and claims. Fredriksson and Tritter [7] argue that the patient perspective contributes with experiential knowledge and focuses on individual/or a specific patient group’s preferences and needs, while the public contributes with collective perspectives and mainly focuses on the general, disinterested, citizen preferences. McCoy et al. [8] state that patient involvement can be used to inform both policy and research based on the experiential knowledge of a condition, while members of the public are involved as members of a relevant population, for example a city, an age group, or a population served by a health system.

Patients and members of the public can be involved individually as well as collectively. It is most common with patient involvement at the individual level and, hence, most studies of PPI impact focus on measures such as clinical outcomes, self-management, or satisfaction with treatment. There is much less evidence of the outcomes or impact of collective involvement, in particular involvement of members of the public in healthcare decision-making [9]. The studies that focus on public involvement find some evidence suggesting that it enhances awareness and knowledge among lay participants but, in general, the evidence of the impact of public involvement in healthcare is scarce and instrumental benefits poorly documented, in particular effects on policy and organisation [9–12]. Such effects include, for example, input in decisions, priorities, policies etcetera, service reorganization and information development and dissemination [9].

### Citizen participation

Research into the functioning and effects of public involvement activities is also found in the vast political science literature on *citizen participation *in policy areas such as environmental policy, urban planning, and public transport. In many systems with representative democracy, it is now a consensus that citizens should be able to participate in decisions that affect them, also between voting in national or local elections [13]. Participatory activities, however, can be conducted in different ways depending on who participates, how participants communicate, and how it is connected to policy action [14]. These activities can take the form of public hearings, neighbourhood councils, citizen juries, consultative commissions, participatory budgets and so on, with a broad distinction being made between activities aiming for opinion formation and decision-making, respectively [15]. Not all participatory activities are suited to serve the same values or have the same benefits, e.g., to incorporate public values and preferences in decision-making, generate more empowered and knowledgeable citizens as political actors, fostering trust in institutions, reducing conflict and achieving more efficient services [16, 17]. In this regard, Fung [18] makes a distinction between the effects of participatory activities on legitimacy, justice, and effectiveness of democratic governance.

Although there are strong normative arguments for citizen participation, empirically, the impact is ambiguous. The impact is mostly evident on the individual level (developmental benefits) and research has focused on the educational function of citizen participation (where the participants increase their knowledge and civic skills and become more competent and confident in their ability to influence policy) and on the integrative function (where participants increasingly feel they are part of the community, also feeling more responsible for decisions). Much less is known regarding the impact on policy and decision-making, i.e., the instrumental benefits of citizen participation [13, 19]. For example, research on citizen juries suggests that it is unclear to what extent they succeed to deliver recommendations usable in policy and practice [20] and there is also a critique against the lack of outcomes of deliberative polling [21]. Similarly, effects of deliberative minipublics are inconclusive, making it unclear if this is an effective method to tackle democratic problems [22]. In addition, support for citizens’ assemblies/deliberative minipublics is partly driven by expectations of a favourable outcome rather than by the institution itself and its collective benefits [23]. However, there is a growing body of literature on coproduction and coordination through voluntary work and the inclusion of non-profit organisations, which suggests that citizen participation at the local level can lead to more effective policy outcomes [24, 25].

Thus, the benefits of participatory activities in representative democracy is not undisputed, even called ambivalent [26]. Although citizen participation arose to overcome problems in representative democracy by engaging citizens in public decision-making between representative elections [15, 17], in practice there is still great uncertainty on the role of this type of participation. Fung [14], for example, argues that it must be seen as a complement to political representation rather than an alternative. It allows policymakers to include new actors in a policy network [26], but it is just one type of input in a policy process or decision [27]. Political representatives must weigh a range of factors when making decisions, some of which might contradict public opinion [27], and accordingly, it is argued it would be problematic if decisions made by a group of citizens would be binding for the larger community [26]. In line with this, Lund et al. [28] argue it is important that there is transparency on how the results from participation activities are to be translated into policy (often the task of civil servants/public managers, which have a major influence over the process and outcome) [29] and because participation is being conditioned by the representative system, it is more likely that influence over policy will take place if it does not challenge the policy-makers’ opinions or the general political agenda. Ideas of representation and accountability seem to make it especially hard to combine citizen participation and representative decision-making [30]. Overall, studies suggest that there is a variation in how much input ordinary people prefer citizens to have over local policy-making processes, but most prefer a decision-making model where citizens and local governments have equally much power, i.e. a combination of representative and participatory approaches [31].

### Citizen dialogues in healthcare: paradoxical democratic consequences?

Although it is not fully clear how citizen participation fits with representative democracy, it has been incorporated at national, regional, and local levels of government in Europe [32]. In this study, we focus on citizen participation in healthcare, more specifically *citizen dialogues *carried out in the Swedish regions (being similar to activities such as citizens juries and deliberative minipublics, but less standardized and uniform). Sweden started a development toward increased citizen participation later than many countries [28]. The slow start for citizen participation in Swedish welfare services can partly be explained by the strong tradition of representative democracy, where political parties and mass movements have been central for participation, also at local (municipal) and regional levels of government [33]. Both the regions—with responsibility for funding and provision of healthcare—and the municipalities—being responsible for social care and elder care, for example—are self-governing and separate elections are held every four years. Thus, regions, as well as the municipalities, have a dual purpose in that they are arenas for service delivery as well as arenas for democracy at the sub-national level [34]. Citizen dialogues (CDs)—which is an umbrella term used in Sweden for a range of participatory activities—is one way of increasing citizen participation in regions and municipalities. CDs are defined as activities aimed at issues that are possible to influence and where decision-makers are influenceable [35]. It should be noted that CDs are not the same as having a user or a patient dialogue, as these specifically target a group of service users and typically aim to gather perceptions and needs of this service among its users. CDs, on the other hand, take a broader approach, were politicians invite various groups or segments of the public to engage in discussions on a health system-related issue [35, 36].

Empirical studies of CDs in Sweden have mainly focused on the municipal level, where CDs are more common, and less on the regions [16, 28, 37]. Studies in the municipalities suggest that the relation of CDs to representative democracy is unclear, that the effects on decision-making are unclear, and that CDs can have paradoxical democratic consequences [16, 28, 36, 37]. According to Tahvilzadeh, the lack of positive outcomes is due to a process that is incoherent, unfair participation, a lack of a common and transparent process, and the fact that some politicians exploit the CDs to legitimate their own policy [16, 36]. Tahvilzadeh argues that if participation is trivial without a meaningful and clear role in decision-making (having a subordinate position) [28], CDs can be harmful to democratic legitimacy, as participants are disappointed that their involvement did not lead to anything [36]. Notwithstanding, a case study on CDs in one of Sweden’s 21 regions found that citizens mainly viewed participation as something positive and that they thought they could contribute with policy input [38]. The politicians in the region also had a positive attitude toward CDs and stated that they got valuable input. However, in accordance with the results from the CDs in municipalities, the study also indicated that participants were unsure of whether the politicians would use the results from the CDs in decision-making and they also felt unsure if their involvement had any effect on policy [38].

To put the case study from one of the regions in a broader perspective, in this article we draw a more comprehensive picture of citizen participation through CDs in the Swedish regions. The aim of the study was to evaluate the functioning of CDs in terms of representation, process, content, and outcomes. The study was carried out through a qualitative case design, building on a modified version of the Abelson et al. [39] analytical framework for evaluating public involvement in healthcare. It contributes to the understanding of public involvement practices in health systems that are politically governed at the sub-national level.

### The Case: Citizen dialogues at the regional level in Sweden

Sweden has a health system that relies on general taxation and universal rights. It is a national health service system, but highly decentralised. The system is based on a model were the responsibility for healthcare is divided between three levels of government [40]. The Swedish state is responsible for overall healthcare policy, while responsibility for funding and provision of healthcare lies primarily with the regions (the municipalities are responsible for care for elderly and disabled) [41].

The regions are largely self-governing and have extensive freedom in how to organise healthcare services [41]. The regions are led by democratically elected politicians and the highest decision-making body is the region council assembly, which appoints a regional board that leads and coordinates the work within the region. The assembly also decides which committees, consisting of elected representatives responsible for a specific policy area, the region should have (SALAR 2021), e.g., the healthcare committee, the regional development committee, or the culture committee. The healthcare committee is usually responsible for planning and ensuring that the healthcare needs of the region’s population are met, thus being the most crucial committee.

In practice, this means that the regions can independently choose how to organise and carry out public involvement [42]. The Municipal Act (2017:725) states that political committees in the regions should strive to consult service users [43]. Since 2006, the Swedish Association of Local Authorities and Regions (SALAR)—an organisation that speaks for municipalities and regions in dialogue with the Government, the Swedish Parliament, government agencies and the like—has worked actively in promoting citizen participation in decision-making in the municipalities and regions, in particular CDs [35]. SALAR produces information and support material and carries out workshops on how to perform CDs. However, it is still voluntary for the regions to decide if, how, and why they choose to involve citizens and how to organise and structure CDs if they choose to conduct them. Data from Statistics Sweden show that less than half of the regions conduct CDs compared to about three out of four municipalities [44, 45].

## Method

### Design

This study was conducted by using a qualitative case design focusing on CDs at the regional level in Sweden [46]. Based on a survey by Statistics Sweden, only nine out of 21 regions stated that they had conducted CDs during the years 2014–2018 (no more recent statistics are available for the regions) and five of these regions stated that they had conducted four or more CDs. We selected to analyse these five regions, and thus included the majority of CDs in the Swedish regions. One of the five regions had to be excluded from the study because it stated that it did not have any saved documentation from the conducted CDs. Inclusion criteria for CDs were that they concerned healthcare in the region and were conducted in the last ten years (2012–2021). To be included, the CD had to take place face-to-face (excluding surveys and digital questionnaires), involve a general or specific section of the public discussing some aspect of the health system in their role as members of the public. CDs that targeted a specific patient group discussing their own care were excluded. The four regions included in this study all have different ways of conducting CDs and different characteristics in terms of geography and demography (see Table 1).

**Table 1:** Characteristics of citizen dialogues in the four studied regions

Region	Population	Size (Km2)	Dialogue organisation	Dialogue strategy	Dialogue projects in the study
**Region A**	> 300,000	< 10,000	The region has six local political committees working in cooperation with the municipalities on social sustainability. Each local committee conducts CDs. Each local committee has their own public manager supporting the CD	The local committees work with the citizen perspective and have an assignment to produce knowledge-documents mainly for development and cooperation	2
**Region B**	> 400,000	> 10,000	At the time of the studied CDs, the region had five political preparation groups for user dialogues assigned by the political healthcare committee. Public managers supported the CD. Now the region has a new organisation	The political preparation groups were assigned on a yearly basis to conduct CDs to gain knowledge about citizen needs. They mainly conducted user dialogues, but sometimes CDs, depending on what needs they were interested in	5
**Region C**	> 1,000,000	> 10,000	Each political committee is responsible for conducting CDs. The region has a central support function with a unit with public managers supporting the CDs	Each committee is responsible for conducting a CD and can ask the support function for help	3
**Region D**	> 1,000,000	> 20,000	There are five local political healthcare committees each conducting CDs. Each committee has their own public managers supporting the CDs	The local healthcare committees are assigned to conduct CDs to identify needs in healthcare	15

Sources: Text about organisation and strategy is based on information from the interviews with public managers in each region

### Data collection

The data consist of official documentation and interviews. The Swedish Ethical Review Authority gave an advisory statement (2019–04650) that a full ethical review was not necessary because the study does not involve collection or processing of sensitive personal data (Law 2003:460). The documents were collected by contacting the regions and asking for documentation from conducted CDs. Contact persons were first contacted by e-mail in early 2021 and received information about the study. They were asked for help finding relevant documentation. The documentation included in the study was public documents produced by the regions to summarize and evaluate the CDs. The documents were utilized as research data to contribute to the knowledge base [47]. As there is no formal requirements that regions perform CDs, and no guidelines for how to document CDs, the documents were heterogeneous both between and within the regions. There were reports summarising many CDs, as well as descriptions of a single CD. To organise the material, the CDs were structured into CD projects where each CD project could describe one or more CD activities. A CD project is the overall mission to conduct CDs on a specific topic, for example mental health. The CD activities are the specific dialogues held in the CD project, for example focus groups or interviews. The regions are referred to as Region A–D.

Document analysis is a systematic procedure for reviewing or evaluating documents. Advantages are for example availability, exactness and coverage. Documents can among other things provide context and be used to track developments, and can be utilized in conjunction with other qualitative data to achieve triangulation [47]. In this study, interviews were primarily conducted to complement and validate the information obtained from the public documentation. Additionally, the interviews were conducted to analyse categories within the framework that were not attainable from the documents alone (see Table 2): thus, they were used to counteract insufficient detail and biased selectivity that may exist in documents. The interviews were held with public managers responsible for conducting CDs within their respective region, i.e., “expert interviews” [48]. An e-mail was sent to the contact person(s) handing out the documents from the regions, with an invitation to participate in an interview. In the e-mail, they received information about the study purpose and that participation was voluntary. If they did not want to participate or did not fit the description of a public manager who had conducted CDs in their region, they were asked to suggest another person appropriate to interview. Because the interviews were seen as a complement to the document study, we interviewed one public manager from each region, and informed consent was obtained from all of them. The interviews lasted between 30 and 38 min and were semi-structured. The interview guide was based on the analytical framework, but also inquired about the organisation and structure of the CD procedure in the region (See Table 1). The quotes from the interviews are cited based on the interview number (interview person (IP) 1–4). The numbers are not connected to the Region letter to make sure that the public managers cannot be identified.

**Table 2:**
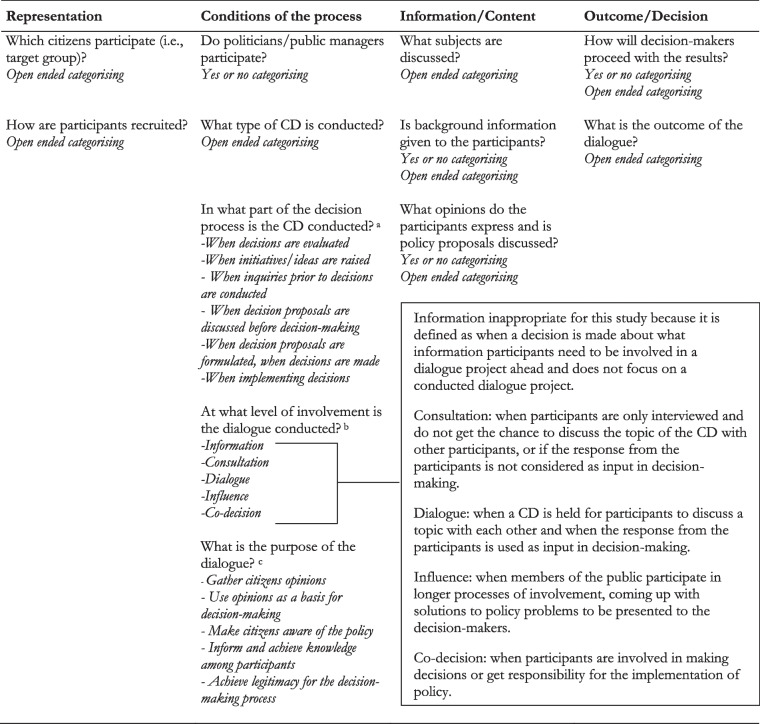
Analytical framework for analysing citizen dialogues

Modified framework for CDs based on Abelson et al. (2003) [30]

^a^Categorising guided by a survey conducted by Demokratiutredningen and SALAR (2015) [40]

^b^Categorising guided by SALAR ladder of involvement inspired by Arnstein [26]

^c^Categorising guided by a survey conducted by Gilljam, Karlsson & Sandell (2010) [41]

### Analytical framework

The analytical framework draws on Abelson et al. (2003) four general principles that can be used to guide the evaluation of public involvement processes in the healthcare sector. These principles are: (i) representation; (ii) procedural rules; (iii) information; (iv) the outcomes and decisions arising from the process. To match the purpose of this study and the previous research on CDs, an adaption of these principles was made. First, we changed the information principle to not only focus on information but also the content of the CDs in general (e.g., discussed topics and expressed opinions). The procedural rules principle was also modified and defined as the conditions of the process. This change was made because we did not want to focus exclusively on rules of the process but the conditions in general, for example the level of involvement and the purpose of the CD. All principles have been adopted to the case of CDs and Table 2 summarises all operationalisations of the principles used to analyse the material. To categorise in what part of the decision process the CD was conducted, the level of involvement with and the purpose of the CD, we used categories developed in other studies, which are presented below the framework in Table 2. The CD project can be categorised in multiple categories, for example both have the purpose to inform and achieve knowledge among participants and achieve legitimacy for the decision-making process.

The framework guided a deductive content analysis of the documentation and interviews. This type of analysis is suitable when the analysis is structured and operationalized on the basis of previous knowledge [49].

## Results

In total, 25 CD projects from four different regions were analysed (two from Region A, five from Region B, three from Region C, and fifteen from Region D). The CD projects sometimes consisted of multiple activities and different types of dialogues and sometimes just a single activity. One CD project in Region C, for example, included three group interviews, four deep interviews and four targeted visits to school classes. In Region D, it was more common that a CD project involved a single activity. These differences can potentially be due to the regions’ different ways of reporting on their CDs. In Region D, the documentation generally consisted of short summaries from a single activity presented on the web site. In the other regions, it was more common that a CD project was summarised in a comprehensive report. The CD projects were conducted between the years 2013 and 2019. Most dialogues were held between the years 2015–2016 (15 out of 25 CD projects).

The results from the study are presented in accordance with the four categories in the analytical framework (see Additional file 1 for results from the document study).

### Representation

#### Which citizens participate?

It was rather common not to target any specific group and to select members of the public within the region (in eight out of 25 projects). Those who sought participation from the public in some cases stated that they aimed to reach a cross section of the public. For example, in a CD in Region B, age and geographic spread in the region was considered (3. Region B). Some CDs focused specifically on age, both by reaching out to younger persons and their parents and by targeting older people. Other groups that were targeted were people of low socioeconomic status or immigrants. Sometimes these groups were referred to as “hard to reach groups.”

#### How are participants recruited?

Many methods were used to recruit participants. If the aim was to reach the public, a broader recruiting campaign was often used. When having a specified targeted group, recruitment was more directed. For example, in Region C when trying to recruit young persons, they contacted youth coordinators in the municipalities to get outreach help (9. Region C). The interviews confirm that many methods were used when recruiting participants, for example by advertising, information letters, population registers, contact persons, and social media. One public manager stated that there is no person that is hard to reach if using the rights methods, for example through libraries, student unions, open houses for seniors, or internet cafés. A couple of the interviewed, however, described that it was difficult to recruit participants to the CDs. One of them thought that it was difficult because it is not as self-evident in what way the citizen is affected compared to a patient dialogue where the participants can influence and improve their own care. It was expressed like this:

“Spontaneously, I would say that it is harder to recruit people to a citizen dialogue compared to a patient dialogue. Because in a patient dialogue you have a purpose to participate as you can improve your own future care /…/ Then you want to be part and improve care to a higher extent. As a member of the public, it might be harder to see your part in it and why you should be involved. (IP3)”

### Conditions of the citizen dialogue process

#### Do politicians/public managers participate?

In the documentation, it was mentioned that politicians were present during twelve of the CD projects. In one case, no politicians or public managers participated, but there was a consult firm who had been assigned to conduct the dialogue (10. Region C). All interviewed public managers mentioned that politicians were usually part of the CDs and that public managers acted as a supporting function. However, the role of politicians and public managers and their relationship differed depending on project and region. For example, two of the interviewed public managers said that they mainly work to support the CDs by taking notes and writing reports, and that the politicians are the ones having the dialogue with the members of the public.

*“It is actually the politicians who do the work, while the public managers do the background work of taking notes and writing reports. The politicians conduct the interviews and conversations, and are involved in analysing the results, after that the public managers write the report based on what they concluded. (IP3)”*

One respondent instead stated that politicians did not participate when meeting with organisations or service providers, but often when meeting with individual members of the public. Another interviewed public manager said that the politicians are the ones initiating the dialogues but that the public managers often design and conduct the dialogue activity and inform the politicians about it afterwards.

#### What type of dialogue activity is conducted?

The most common type of CD activity was discussion groups or focus groups (12 out of 25). During the group discussions, different methods were used to activate the participants and to get their views. Other discussion types were workshops, meetings, speed interviews, and group interviews. Another usual type of CD was to conduct a directed visit to a strategically chosen site. Examples were markets, festivals, school classes, or civil society organisations. It was also common that CDs were carried out as interviews instead of group discussions, in which the participants did not have the chance to discuss topics with other participants. Some of the CD projects were carried out as surveys or digital surveys, but these activities were not included in the study.

#### In what part of the decision process is the dialogue conducted?

Most of the CDs were held when initiatives and ideas were raised early in the decision process (in 19 out of 25 projects). For example, in a CD project in Region B, the aim was to gather the citizens’ views on how self-care and health promotion could be improved (3. Region B). Three projects were conducted when inquiries are made before a decision, for example, a case in a CD project conducted in Region A where it was stated that the participants’ thoughts on healthcare needs were to be used as input for the decision on the new strategy for future healthcare services in the region (1. Region A). Three projects were conducted when a policy was evaluated. For example, in Region D when a directed visit was made to a family centre to explore what the visitors thought about the centre (13. Region D). No project was conducted in the process of drafting a decision or when a decision was made. Four CD projects were not described as being part of the decision process at all.

#### At what level of involvement is the dialogue conducted?

Six of the CD projects were categorised as *consultation*. One example is a CD in Region D where region representatives met with participants, but where the participants did not get the chance to discuss issues with other participants (23. Region D). The most common level of participation was *dialogue* (11 out of 25 projects). One example is a CD project in Region A where 24 discussion groups were conducted to use the citizens’ thoughts as input in decisions about the region’s future healthcare strategy (1. Region A). Only one of the CD projects was categorised as *influence,* a CD project in Region C, which was about future healthcare within the region (9. Region C). In this CD, the participants were involved in a longer process with multiple meetings with the purpose to present the solutions from the dialogues to the decision-makers on the healthcare committee. None of the CD projects reached the *co-decision* step, as none of the CDs involved the participants in making decisions or let them take responsibility for the implementation of policy. Eight of the CD projects could not be categorised due to lack of information.

#### What is the purpose of the dialogue?

The CD projects often had several aims. The most common purpose was to gather citizens’ opinions on a specific or general matter (in 18 out of 25 projects), such as collecting thoughts from members of the public on their healthcare needs (1. Region A). It was also rather common that the purpose was to use the views of the citizens as input in decision-making (ten out of 25 projects). For example, in a CD project in Region B, the purpose was to create a decision-basis for a future strategy for primary care services (5. Region B). In some of the CDs, the decision-makers were not only interested in the views of the citizens but also aimed to inform them about a specific matter (four out of 25 projects), such as the future challenges for healthcare (9. Region C). Some CD projects stated that the aim was to make citizens aware of the politicians’ role and their policies (three out of 25 projects). For example, in a dialogue in Region D, it was stated that one of its aims was to inform the citizens about the role and responsibilities of the healthcare committee (11. Region D). In a few projects, the aim was partly described as creating democratic legitimacy by involving citizens in the decision-process (in four out of 25 projects). Five of the CD projects could not be categorised due to lack of information.

In the interviews two main purposes were mentioned. Firstly, that the CDs aim to improve the basis for decision-making and collect views on needs, as well as suggestions for improvements to be used by politicians in improving healthcare. The second aim was described as a democratic value of creating legitimacy in the decision-making process by involving citizens and giving them an opportunity to be part and to be able to exercise influence.

### Content

#### What subjects are discussed?

The subject in seven out of 25 CDs was healthcare services in general, as in Region A when discussing the future healthcare strategy (1. Region A). The rest of the CD projects focused on specific aspects of healthcare such as equal care, child healthcare, mental health, e-health, primary care, illness among young, and cooperation between care levels. For example, in a CD project in Region C, the focus was citizens’ views on e-health and communication with the healthcare services (10. Region C).

#### Is background information given to the participants?

In ten of the CD projects, it was stated in the documentation that information about the CD or the topic was given in advance. For example, in a CD project in Region B, participants were given information about the concept of e-health before discussing their thoughts on the topic (7. Region B). In the interviews, however, it was indicated that background information was generally given about the CD project and the topic to be discussed.

#### What opinion do the participants express and is policy proposals discussed?

In 18 of the 25 CD projects, there were discussions about policy proposals. These proposals were often broad, as in a CD in Region C where participants suggested ways to involve younger persons in society by discussing everything from how to get a job to how to stop young people from taking drugs (9. Region C). This can be compared with a dialogue in Region B where specific suggestions to improve primary care were presented, for example ways to make it easier to book an appointment through the web page, to increase the availability of specialised doctors, and to make it easier to make complaints (5. Region B).

### Outcomes

#### How will decision-makers proceed with the results?

In eleven of the 25 CD projects, it was stated how the results from the dialogue would be used in future decision-making. Many of the CD projects specified that the results would be used as input in political decisions. None of the CD projects however stated *how* the results would be used more specifically. In some CD documentation, it was stated that the results would be presented to the healthcare committee. The conductors of the dialogues sometimes gave their own advice and recommendations in the written reports about what decisions should be made based on the results from the dialogues. For example, a CD project in Region C included 13 specified recommendations to the region on how to involve younger people in society (9. Region C).

In the interviews, all public managers claimed that the results from CDs are used in decision-making. However, the routines differed between the regions. Two of the region representatives described that the results often end up in reports that are presented and discussed in the healthcare committee. One public manager highlighted that the region should improve the documentation and, in a more structured way, include experiences and conclusions from the CDs in the regions’ policy documents, needs assessments, and documents specifying priorities and goals. One of the region representatives, however, described a structured routine where the CDs are part of the decision-making process and the recommendations from the CD reports are supposed to be used when writing assignments to the providers in the rulebook for primary care, agreements, and contracts with care providers. However, this was described as difficult because CDs are more general than patient dialogues:

*“The recommendations become more general, to society, for example that schools should have information about tooth brushing (IP3).”*

#### What is the outcome of the dialogues?

As the documents only summarised the results of the CDs, they could not be used for analysing the outcome or impact of the dialogues in policymaking. In the interviews, the public managers’ perceptions of impact were somewhat ambiguous. They simultaneously stated that the CDs led to impact in the decision-making process and gave examples of such impact, and said they were unsure of the impact. One example of impact was when a CD report became part of the decision-basis for a regional development strategy. Another example was when a CD at a family centre showed that it was hard to reach some parents, which led to the healthcare committee starting an investigation around this. An example of disappointing outcomes was also given by a public manager whom described a CD project on equal care that did not lead to as many intent policy formulations as wished for, but only some general ones, which were not only the results of the CD, but were issues that the region already worked with, such as equal treatment, patient centred care, and a right to an interpreter in healthcare if you are born abroad. As mentioned, the representatives also described that they really did not know how much impact the CDs had, as it was not documented and not traceable. For example, one of them described how, after a CD, they always send the summary reports to the politicians, but that it is not obvious how the results are used in decision-making. It was expressed like this:

*“The reports [CD results] are always sent to the politicians and are processed by their support functions, so it always undergoes some sort of political process, but what they do with it and how it impacts decisions is still arbitrary (IP1).”*

## Discussion

The aim of the study was to evaluate the functioning of citizen dialogues (CDs) in Swedish healthcare in terms of representation, process, content, and outcomes. These four aspects of public involvement activities are discussed below and related to the literature on citizen participation in systems based on representative democracy.

Regarding *representation*, the results show that participants in the CD projects were recruited from the public in about every third dialogue (self-selection). It was also common with CDs directed towards young or old individuals, but also immigrants or unemployed (selective recruitment). Thus, there was a mix between involving citizens more broadly, which implies a higher risk of skewness (e.g., towards a higher level of education [14]), and involving a more targeted group of citizens. As pointed out by Slutsky et al. [4], there is often an ambiguity as to whom public participants represent and if they participate in the role of patient, citizen, or consumer. In some CDs, the targeted groups were selected because politiciansy are likely to consume a specific type of care and in other ones because they are part of a group that is “hard to reach” and have a weaker voice in the democratic dialogue. Thus, some of the CDs can be seen as an attempt to counteract political inequality by inviting groups that are less represented among elected politicians [14], while the dialogues targeting the general public might be a way to increase legitimacy by reducing the distance between policy-makers and citizens and making policies more grounded [14]. Also, however, the “hard to reach” participants were partly seen as consumers sharing information about their care needs, implying that these CDs also aim to improve the quality of particular services, i.e., enhance the effectiveness by more closely matching the values, needs, and preferences of certain citizen groups [14].

Regarding the *process*, the results show that politicians often were involved in the CDs (supported by public managers taking notes and producing reports), which signals that they are invested in the process, which is a precondition for the CD to be successful, as they are the ones making the final policy decisions [35]. However, earlier research has also shown that public managers’ attitudes towards citizen participation is important for the outcome, not least because they constitute the link to the politicians and are a primary source of information [29]. Based on the interviews with the public managers, they all found CDs as a valuable tool for public participation in the decision-making process. Similar to studies on CDs in the Swedish municipalities, where politicians stated that they found CDs more important in the beginning of the decision-making process compared to the end of it [50], the CDs in Swedish healthcare were most often held in the beginning of the decision-making process, and the purpose of the CD projects were generally to use the results from the dialogues as input in decision-making and to gain knowledge (i.e., consultation and dialogue). This gives the participants an opportunity to affect policy early on, which is usually described as a success factor of PPI [29, 51]. However, too early involvement—sometimes even unrelated to a specific policy process—could make citizen input too vague or broad, in particular if members of the public are asked for their opinions on a broad topic, such as how to improve public health. In fact, the *content *of most CDs was healthcare in general, although some focused on specific issues such as primary care, mental health, e-health, and communication. Too broad of topics, without scenarios to discuss or prioritise between (i.e., preference expression rather than preference development) [14], risk leading to watered down or unspecific suggestions or policy input that is of no real use for the decision-makers. One such example from our study was the recommendation that schools provide information about tooth brushing. This may emphasise drawbacks of participation, such as creating more bureaucracy and slowing down decision-making [27]. One strategy to reach more specific policy suggestions is to develop preferences among the participants. This can be carried out by providing background information and educational material and then discussing merits and trade-offs between different solutions [14]. In a majority of the CD projects, it was not stated if any background information was given and, even if the public managers stated that they informed the participants to some degree about the project, it does not seem to be used to develop preferences.

Results regarding the *outcome* show that many CD projects did not specify *how*the results from CDs would be used in decision-making. Even if the interviewed region representatives were sure that results from CDs were used in decision-making, and could give some examples of impact, they described a problem in tracing the outcomes from dialogues. The regions had different levels of formalisation for inclusion of CDs within the decision-process but most expressed a need to become better at documentation and follow-up on the outcomes. Missing evidence of impact and poor documentation of effects is in accordance with previous studies of public involvement [9, 10]. Lack of evidence of impact can be seen as a problem, but Conklin et al. (2015) state that too much focus on outcomes risks missing out on the normative argument for public involvement as something good in itself as part of the democratic process (improving justice and legitimacy [10, 14]). In line with others, they therefore argue that public involvement should not be solely evaluated based on the impact of the activity, but also on the quality of the procedure. Similarly, Lowndes et al. [27] argue that what is most important to know when evaluating participation is whether the policy-makers have given the results from participatory activities due weight, which we cannot answer in this study.

Further, Thurston et al. [52] argue that evaluating public involvement by only looking at the impact on decision-making does not capture the effect that a public involvement activity might have. This argument is based on the view that the policymaking process is not linear or rational and is influenced by multiple factors. By viewing policymaking as a political sphere, where public involvement can affect the political space in different contexts, the effect of public involvement becomes more complex. Public involvement can affect the problem formulation as well as identification of solutions, which can lead to political change. Even if no decision is made based on a CD or similar, public involvement can still influence the problem and policy agenda. A counter argument to this view is that impact and documentation of impact is an important part of democratic procedure. Tahvilzadeh states that if there are no reports of impact or input from CD projects on decision-making, they risk becoming trivial [16]. Then the process risks leading to less democratic legitimacy, as the participants feel disappointed and excluded instead of included. This is an apparent concern with the CDs in the Swedish regions, and triviality (i.e., limited scope and powers of participatory activities) has been identified as one of three challenges to creating successful participatory governance; the others being absence of systematic leadership and the lack of popular or elite consensus on the place of direct participation [18]. One indication of the latter is that it was perceived as more problematic to recruit participants to CDs compared to patient dialogues, likely because the focus of the CDs is broader and not necessarily on services used by the participants or located in their nearby area. A possible solution could be to change the level of dialogue to the local or community level, for example by recruiting participants from a community when establishing a new health care centre. This might however be problematic in Sweden as healthcare is politically governed at the regional level and not locally in the municipalities.

### Limitations

There are some limitations to this study. First, we had to rely on the regions’ own CD documentation (available at their websites or after communication with public managers), which varied in form and detailedness, both within and between the regions. This means that some information was lacking and that not all aspects of the CDs could be examined. It is possible that some CD projects were overlooked due to lack of documentation or guidance on where to find the documentation. Furthermore, another issue relates to the discrepancy in the number of retrieved CD projects from the regions, ranging from two to 15 CD projects. To address this discrepancy, we had to consider the dominance of the region with 15 projects when presenting the results.

Second, only one interview was conducted within each region. However, the interviews were a complement to the main data source (the CD documentation material), and in each region we interviewed the public manager with the most insight into the CD process. As the interviews were a complement, they gave additional information about the region’s strategy, organisation, and outcomes of the CDs, and helped validate the findings from the document study. Another limitation is that we chose to only interview public managers and no politicians or public participants, which are likely to have other perspectives [48]. We, however, argue that the public managers who support the CD projects and document them are the most suitable to give information about the aspects we were interested in. Their crucial position has been described by Lund et al., [28] who argue that all input from CDs must be translated into a written document, and this translation gives public managers significant influence over what input reaches the policy process. Their central position could, however, entail a will to convey a picture of CDs in their own region as successful. This was, however, not our impression from the interviews. A potential final limitation is that the collected documents were retrieved from CDs conducted between the years of 2013–2019, while the interviews were conducted in 2021. This time difference may pose a challenge in accurately recalling the details of CDs conducted several years prior. However, our interpretation is that the respondents provided credible accounts of how the CDs were conducted, which aligned with the documentation from that period.

## Conclusion

In sum, this study has provided a more comprehensive understanding of the functioning of CDs in the Swedish regions (i.e., in the decentralised Swedish health system), which appears to resemble that in the Swedish municipalities. At both sub-national democratic levels, it is still unclear how input from CDs is (to be) used in policymaking and thus unclear how this type of citizen participation interacts with representative democracy. Is it supposed to enhance legitimacy, justice, or effectiveness in governance; goals that sometimes conflict with each other? Furthermore, the analysis of the CDs points to a risk of triviality, either that policy input becomes too general as a response to too broad of questions or that the input from the dialogues is not given enough weight, also suggesting that it is more problematic to find relevant ways to involve members of the public in healthcare development than patients with experience from treatments or services. However, to further the understanding on the impact of CDs, we need to know more about how CDs are taken into consideration and how much weight they carry in relation to other types of information that politicians use, and in relation to other types of PPI. Behrer & Breux [53] argue that different participation activities can be difficult to reconcile and can lead to a competing situation, and it is important to notice that CDs are not the only participatory activity taking part within the regions. For example, the regions also conduct patient dialogues, have senior citizen councils, and opportunities to come with written feedback on services.

### Supplementary Information

**Additional file 1.**

## Data Availability

Part of the dataset is publicly available from the four regions (the CD documentation). The interviews are not publicly available due to ethical reasons and the right to confidentiality for recorded persons but are available from the corresponding author on reasonable request.

## Abbreviations

CD: Citizen dialogue
PPI: Patient and public involvement
SALAR: The Swedish Association of Local Authorities and Regions
IP: Interview person
