# Supplementary material for: Public involvement in the Swedish health system: citizen dialogues with unclear outcomes
Source: BMC Health Serv Res. 2023 Sep 4;23:947. doi: 10.1186/s12913-023-09947-x (PMC10478364; doi:10.1186/s12913-023-09947-x)
Supplement: Supplementary file 1 — Additional file 1. [file 12913_2023_9947_MOESM1_ESM.pdf]

Supplementary file 1. Characteristics of the citizen dialogues (CDs)

Region<br>Year	Type of material	Topic	Represented groups	Type of dialogue	When in decision-process?	Purpose	Level of participation	Description of how results will be used
1. Region A<br>2016	Concluding report from several CDs	Healthcare in general	Young, mid-life, elderly	24 discussion groups	When initiatives and ideas are raised.<br>When investigations prior to decisions are conducted	Gather citizens opinions, use opinions as a basis for decision making	Dialogue	Yes
2. Region A<br>2018	Concluding report from several CDs	Mental health	Young, young adults, staff working with young people	No description of activity, but 287 individuals participated in 63 dialogue group meetings	When initiatives and ideas are raised<br>When investigations prior to decisions are conducted	Gather citizens opinions, use opinions as a basis for decision making	Dialogue	Yes
3. Region B<br>2017	Concluding report from several CDs	Health promotion	General public	8 focus group conversations	When initiatives and ideas are raised	Gather citizens opinions, use opinions as a basis for decision making	Dialogue	No
4. Region B<br>2016	Concluding report from several CDs	Equal care	Low socio-economic status, parents	No description of number, but of activities: individual interviews, quick interviews and a reference group	When initiatives and ideas are raised	Gather citizens opinions, use opinions as a basis for decision making	Dialogue	Yes
5. Region B<br>2015	Concluding report from several CDs	Primary care	General public	8 focus group conversations	When initiatives and ideas are raised<br>When investigations prior to decisions are conducted	Gather citizens opinions, use opinions as a basis for decision making	Dialogue	Yes
6. Region B<br>2015	Concluding report from several CDs	Equal care	Low socio-economic status, parents	9 interviews individually or in group, 28 quick interviews	When initiatives and ideas are raised	Gather citizens opinions	Consultation	No
7. Region B<br>2014	Concluding report from several CDs	E-health, communication channels	General public	12 focus groups at directed visits	When initiatives and ideas are raised	Gather citizens opinions	Dialogue	Yes

8. Region C 2018	Concluding report from one CD	Healthcare with a focus on cooperation	General public	1 discussion group	When initiatives and ideas are raised When decisions are evaluated	Gather citizens opinions, use opinions as a basis for decision making	Dialogue	No
9. Region C 2013	Concluding report from several CDs	Healthcare in general	General public	28 discussion groups	When initiatives and ideas are raised	Gather citizens opinions, use opinions as a basis for decision making, inform and achieve knowledge among participants	Influence Dialogue	Yes
10. Region C 2019	Concluding power point presentation from several CDs	E-health, contact with the health care service	Older, newly arrived (from other countries), young	3 group conversations, 4 deep interviews, 4 directed visits	When initiatives and ideas are raised	Gather citizens opinions	Dialogue	No
11. Region D 2016	Short summarized information from one CD	Healthcare in general	Newly arrived (from other countries)	1 directed visit with discussion in groups	-	Gather citizens opinions, make citizens aware of the policy, inform and achieve knowledge among participants	Dialogue Consultation	No
12. Region D 2016	Short summarized information from one CD	Child care	Parents, staff working with child care	1 directed visit with group discussion	When decisions are evaluated	-	-	No
13. Region D 2016	Short summarized information from one CD	Child care	Parents	1 directed visit with group discussion	When decisions are evaluated	-	-	No
14. Region D 2016	Short summarized information from one CD	Healthcare in general	General public	1 discussion group	-	-	-	No
15. Region D 2015	Short summarized information from one CD	Healthcare in general	General public	1 directed visit to an event	-	Make citizens aware of the policy, inform and achieve knowledge among participants	-	No
16. Region D 2015	Short summarized information from one CD	Healthcare in general	General public	1 directed visit to an event	-	Make citizens aware of the policy, inform and achieve knowledge among participants	-	No
17. Region D 2016	Short summarized information from one CD	Communication channels	Young people with disability and mental illness	-	When initiatives and ideas are raised	-	-	No
18. Region D 2016	Short summarized information from one CD	Communication channels	Older people	1 discussion group	When initiatives and ideas are raised	-	-	No
19. Region D 2015	Short summarized information from one CD	Healthcare in general	Young people	-	When initiatives and ideas are raised	Gather citizens opinions, achieve legitimacy for the decision-making process	-	No

20. Region D 2019	Short summarized information from several CDs	Illness among young people	Families, ill young people	3 round-table meetings	When initiatives and ideas are raised	Gather citizens opinions, use opinions as a basis for decision making	Dialogue	Yes
21. Region D 2018	Short summarized information from several CDs	Coordination in healthcare	Parents	4 directed visits to a service provider	When initiatives and ideas are raised	Gather citizens opinions	Consultation	No
22. Region D 2018	Short summarized information from one CD	Coordination in next of kin care	Next of kin carers	1 group conversation	When initiatives and ideas are raised	Gather citizens opinions	Dialogue	Yes
23. Region D 2016	Short summarized information from one CD	Mental health among young people	Young people, parents	1 directed visit to an event	When initiatives and ideas are raised	Gather citizens opinions, use opinions as a basis for decision making, achieve legitimacy for the decision-making process	Consultation	Yes
24. Region D 2016	Short summarized information from several CDs	Mental health among young people	Young, staff working with young people	2 directed visits with group discussions	When initiatives and ideas are raised	Gather citizens opinions, use opinions as a basis for decision making, achieve legitimacy for the decision-making process	Consultation	Yes
25. Region D 2016	Short summarized information from one CD	Emergency care, wrong level of care	Citizens seeking emergency care, family, staff	1 directed visit to a service provider	When initiatives and ideas are raised	Gather citizens opinions, use opinions as a basis for decision making, achieve legitimacy for the decision-making process	Consultation	Yes

- means that there is no information or not enough information to classify the CD.
